# Modeling as a Decision Support Tool for Bovine TB Control Programs in Wildlife

**DOI:** 10.3389/fvets.2018.00276

**Published:** 2018-11-06

**Authors:** Graham C. Smith, Richard J. Delahay

**Affiliations:** National Wildlife Management Centre, Animal and Plant Health Agency, York, United Kingdom

**Keywords:** badger, model, decision making, bovine tuberculosis, simulation

## Abstract

Computer modeling has a long history of association with epidemiology, and has improved our understanding of the theory of disease dynamics and provided insights into wildlife disease management. A summary of badger bovine TB models and their role in decision making is presented, from a simple initial SEI model, to SEIR (inclusion of a recovered category) and SEI_1_I_2_ (inclusion of two stages of disease progression) variants, and subsequent spatially-explicit individual-based models used to assess historical badger management strategies. The integration of cattle into TB models allowed comparison of the predicted impacts of different badger management strategies on cattle herd breakdown rates, and provided an economic dimension to the outputs. Estimates of R_0_ for bovine TB in cattle and badgers are little higher than unity implying that the disease should be relatively easy to control, which is at odds with practical experience. A cohort of recent models have suggested that combined strategies, involving management of both host species and including vaccination may be most effective. Future models of badger vaccination will need to accommodate the partial protection from infection and likely duration of immunity conferred by the currently available vaccine (BCG). Descriptions of how models could better represent the ecological and epidemiological complexities of the badger-cattle TB system are presented, along with a wider discussion of the utility of modeling for bovine TB management interventions. This includes consideration of the information required to maximize the utility of the next generation of models.

## Introduction

Mathematical models are both a simplification of reality and a reflection of our current understanding. As a working hypothesis of our supposed reality they can consequently only be shown to be wrong (1). A good model should only include necessary parameters, although the definition of “necessary” depends on the model's purpose. There are three main types of model: statistical, mathematical and simulation. Statistical models find relationships between parameters and will not be considered here. There is a continuum from mathematical to simulation models, but in general the former are used to investigate how a system works, while the latter, usually mechanistic, can be used to investigate management options.

Bovine tuberculosis (bTB, caused by *Mycobacterium bovis*) is a serious disease of cattle and control can be made more challenging by the involvement of wildlife reservoirs (2). In the UK and Republic of Ireland, European badgers (*Meles meles*) are implicated in the persistence and spread of infection to cattle (3, 4). In both countries management of the risks of transmission to cattle has focused on culling badgers (5, 6). As badgers are native this imposes certain practical restrictions and attracts controversy. There has also been substantial Government investment in recent years in the development of a badger vaccine (7, 8) with small-scale deployment for research and operational purposes (9, 10).

*M. bovis* in badgers is a chronic progressive condition, which can lead to debilitating disease and death, although many infected badgers survive for years and prevalence can average about 10–20% or higher (11). Principal sites of infection are the lungs and associated lymph nodes. Badgers may exhibit a range of responses to infection ranging from latency (host infected but bacteria are effectively contained), to generalized disease (12) when they are likely to be most infectious, potentially shedding bacteria in sputum, feces, urine, or pus from wounds or abscesses (13). Once infectious, onward transmission of *M. bovis* occurs by aerosol transmission among animals in close contact, via bite wounding (14), and indirectly through environmental contamination (15, 16). Transmission to cattle is thought to be through contact with bacteria in the environment rather than via direct contact (17, 18).

Mathematical modeling has a long history with the badger-TB system. This has ranged from modeling the dynamics of infection in badger populations, to complex two host badger and cattle systems, and simulating the impact of management to inform disease control policy (see below). Modeling is often referred to as an iterative process. Models can be used to investigate the theoretical aspects of disease ecology and management, data are investigated to determine parameter values, and the models can determine where the data are deficient. If the model output is sensitive to parameter estimates that are uncertain or poorly measured, then this can be used to define new research questions and hence to guide the collection of empirical data to fill gaps and reduce uncertainties. These new data are then incorporated and the process repeated. This iteration rarely occurs in reality since people who generate empirical data and those who write models often work independently. Our research team (the UK National Wildlife Management Centre and its precursors), are therefore relatively unusual in this regard, being responsible for both the longest field study of badgers and bovine TB epidemiology (19), and the evolution of a series of models describing this system. Since reviews of badger/bTB models are already available [e.g., (20, 21)], we provide a historical narrative of the development of these models, the roles they have played in supporting decision-making, and our perspective on the future of modeling in this complex and challenging area of disease management.

## Historical review

Early badger/bTB models investigated population dynamics in detail since this was the first opportunity to examine data from an ongoing study, resulting in a simple SEI (susceptible, exposed and infectious disease categories) model (22). This work summarized the known information on population dynamics (e.g., fertility and mortality rates). The resultant model suggested that disease induced mortality was 2.5 times natural mortality and thus exerted a high level of population suppression. The model was used to determine R_0_, the expected number of secondary cases produced by one infected case in a completely susceptible population. This is a measure of the transmission potential of a disease and the estimated R_0_ lay between 1.9 and 9.7, which reflected the level of parameter uncertainty. This model also explored pseudo-vertical transmission (i.e., mother to offspring transmission via close contact or ingestion of infected milk), the potential presence of asymptomatic carriers of infection, environmental reservoirs and inactive (short-term non-infectious) cases. With hindsight we can see that consideration of these phenomena illustrates the short-fall in empirical evidence on disease progression at the time (23).

The next model was an SEIR (SEI plus a recovered category) model and a parameter search used to refine population and epidemiological values (24). However, the inclusion of a “recovered” class was not itself tested, and has not been implemented in most other models. A further variant was the SEI_1_I_2_ model which permitted two levels of infectiousness (associated with early and advanced disease) and pseudo-vertical transmission (25). Investigation of six potential model structures suggested that those with two levels of infectiousness had some support.

The construction of an individual-based simulation model permitted the inclusion of territoriality and spatial components (26), which resulted in disease clusters and removed the clear relationship between disease prevalence and population suppression. The use of social groups also meant that the threshold density for disease persistence was now considered as the average minimum social group size that would permit disease maintenance. Although this model also suggested substantial disease-induced population suppression, the effect was reduced by the spatial clustering of disease (26). This was the first model to assess different historical badger management strategies (27): Gassing, Clean Ring, Interim and Live Test strategies (see Table 1 for definitions). Model outputs suggested that the most efficient strategies were Gassing and the Clean Ring since they may remove foci of infection. The model also explored badger vaccination and concluded that it would take between 10 and 30 years to eradicate bTB with a perfect vaccine, depending on the efficacy of delivery. A later version investigated fertility control (through the use of a theoretical oral contraceptive) and concluded that in isolation this would not eradicate bTB in badgers but that disease control was possible when combined with high levels of culling (30). A simple generic model was used to simulate combined vaccination and fertility control and concluded that the reduced efficacy of vaccination, relative to culling, disappeared when allied with fertility control, and thus a combined approach could be effective (31).

**Table 1:** A summary of historical badger control strategies used in England.

**Control Strategy**	**Approach**	**Estimated Efficacy of control^1^**	**Area^2^**
Gassing	Gassing setts where badgers confirmed with bTB.	90%	Up to 10 km^2^
Clean Ring	Cage trap and shoot social groups in an expanding ring where confirmed with bTB	80%	Mean 9 km^2^
Interim	Cage trap and shoot badgers on and around confirmed cattle breakdowns	70%	Mean 12 km^2^
Live Test	Trial strategy of cage trap and shoot in response to an antibody test.	80%	Mean 1 km^2^

*^1^from Smith et al. (28), ^2^from Krebs et al. (29)*.

A revision of Smith et al. (25) was the first model to predict limited population suppression (32), which was supported by the field data (33). This model also suggested that culling lactating females only had a limited impact on disease control, which supported the prevailing policy of releasing them.

A return to a simple model investigated the effects of social perturbation [the process of disruption of the social structure of populations subjected to culling: Swinton et al. (34)], which could theoretically increase absolute numbers of infected animals. Both this and a subsequent study (31) also investigated the effect of fertility control, and suggested that lethal control was generally more effective. However, Smith and Cheeseman (31) found that permanent sterility combined with vaccination could be just as effective as lethal control, and would permit disease elimination without risking population extinction. Using updated parameters, a simple mathematical model of badgers and cattle concluded that R_0_ was lower than previous estimates, at about 1.1 (35), and was supported by subsequent empirically-derived estimates of 1 to 1.2 (11). These findings suggest that control would require less than a 20% reduction in transmission rates to eliminate disease, although this appears to contrast with field experience.

Simulation models then added cattle, firstly as a simple homogenous set of herds connected to each badger social group (36). This model was used to assess the live test strategy (36), and other historical and prospective strategies (28) including vaccination (37). These studies concluded that the use of a live test required better test sensitivity and that more badgers per group needed testing and that Gassing and the Clean Ring were the most effective historical strategies. The model identified proactive widespread vaccination as the most effective vaccination strategy requiring vaccinating at least 40% of badgers every year to eliminate disease and that combined strategies gave the best initial reduction in cattle herd breakdown rates. Since the models were generating results that could inform policy, there was merit in ensuring the results were robust, so a second independent model was developed using the same input data. Reassuringly, this model gave very similar results (38).

Most of the data came from a field study of bTB epidemiology in badgers (39–42). When the latest models were subjected to sensitivity analysis, the outputs were found to be sensitive to the two infectious classes (particularly the more infectious category, and their mortality rates). This led to more detailed field research, which allowed disease categories and survival rates to be refined (43) and incorporated into subsequent models.

Between 1998 and 2005 a large scale field experiment took place in England, to determine the role of badger culling as a means of controlling bTB in cattle. Results of the Randomised Badger Culling Trial (RBCT) demonstrated that cattle herd breakdown rates were significantly reduced within proactively culled areas, but increased around the edges (4). Subsequent investigations identified significant spatial disruption of badger social group territories after culling (44), which tied in with previous observations of post-cull badger populations, including enhanced movement of surviving animals [reviewed by (45)]. The long-term field data from the Woodchester Park study demonstrated a clear link between badger movement rates and prevalence of bTB in an undisturbed population (46), suggesting that enhanced movements of badgers following culling might have adverse epidemiological outcomes. Thus, the model could now be updated by changing badger behavior (movement probabilities) to generate the pattern of herd breakdowns seen in the field. This approach of pattern-oriented modeling had recently been taking root in ecological models (47, 48). In a subsequent model, badger movement was simulated to match data from field studies (45), and the contact rate amongst badgers increased until the simulated rise in the herd breakdown rate matched that observed during the RBCT (49). The revised model also included a more realistic cattle layer incorporating individual farms and cattle movements, allowing investigation of pre-movement cattle testing, and including farm economics so that a partial cost-benefit analysis could be conducted. Even if most of the badger control costs were borne by the farmer the model concluded that, due to perturbation, the cost-benefit analysis was nearly always negative. Preventing badger immigration, or if perturbation did not occur, an economic benefit was more likely than not (49). If the Government bore the cost of badger culling then even without perturbation, most scenarios indicated an overall economic loss (50).

The Smith et al. (50) model was revised and updated with further field data, and used to investigate different bTB control strategies. In Wales the model was used to inform a decision on what badger management approach to take in an Intensive Action Area (IAA) identified by Government (51–53). The IAA was subjected to badger vaccination, and following 4 years of treatment the model was used to determine the effects of a lack of vaccine in the fifth year (54). This indicated that the fifth year of vaccination would add relatively little to the overall benefit, and no discernable benefit if vaccination was delayed by a year. This suggests that, following 4 years of treatment, herd immunity was raised to a level sufficient to justify a break in vaccination effort. In Northern Ireland, simulations investigated selective badger culling to inform proposals for a trap, live test and cull or vaccinate (TVR) approach (55), which is currently being trialed (56). In England the model was used to assess different culling and vaccination policies and concluded that in order to realize a benefit, badger culling would need to continue for at least 4 years and that low culling efficacy or an early cessation to culling could lead to an increase in the number of herd breakdowns (57).

Other models have investigated different selective or combined badger management strategies (58–60). Supporting previous results, these studies indicated that badger culling may reduce disease prevalence, but alone cannot eradicate bTB, and that combined vaccination strategies may be the most effective. None of the models have found that a single strategy is the most effective, generally agreeing that combined approaches are required, together with strong cattle measures. The deployment of such approaches in the field would provide data to test these predictions. The inability of models to easily eradicate bTB with single approach methods contrasts with the available estimates of R_0_, which have suggested that control should be easier to achieve.

Although the principal driver for interest in bTB is to control the disease in cattle, there has been substantially less modeling focused on cattle. However, models of bTB in New Zealand were used to investigate cattle management. These indicated that improved cattle testing (61) and cattle management (62) alone were insufficient to eradicate bTB in the presence of the local wildlife vector (the brushtail possum *Trichosurus vulpecula*). A further model indicated the potential benefits of increased cattle testing, and reduced cattle movement in combination with wildlife vector control (63). These results, combined with output from other models (64–70) were used to inform the eradication strategy (https://ospri.co.nz/our-programmes/tbfree/about-the-tbfree-programme/about-bovine-tb/history-of-tb/).

Other wild mammal species can be infected with *M. bovis* and some may act as maintenance hosts, with potential onward transmission to cattle. In Spain, wild boar *Sus scrofa* and red deer *Cervus elaphus* appear most important as wild reservoirs of infection (71) and in North America white-tailed deer *Odocoileus virginianus* are involved in transmission to cattle (72). A model of bTB in white-tailed deer assessed various vaccination and targeted removal strategies and concluded that vaccination (alone or combined with targeted removal) needed to be undertaken annually to achieve a detectable reduction in prevalence (73), and currently an oral vaccination approach is under investigation (74). However, to date modeling has been applied to a far lesser extent to these situations compared to the badger-cattle bTB system.

The historical evolution of modeling described above clearly indicates where models have been used to inform decision making on bTB control in wildlife. In the badger bTB system, the interplay between field studies and modeling, and the use of models to guide decision making have been particularly prominent. Early models concentrated on increasing our understanding of the system with limited impact on decision making, but derived parameter estimates necessary for later models, which informed further field studies to refine key parameters. Successive models, which have generally included stochasticity, have since played a more explicit role in supporting decision making.

## Recommendations

Below we describe a series of recommendations borne out of our experience of data analysis and modeling largely in relation to the badger/bTB system. Our recommendations relate first to themes for future models of bTB in badgers, and second to the presentation of model outputs to decision makers.

### Future models of bTB in badgers

The following themes could be usefully explored in future models of bTB in badgers, but may also apply to other wildlife disease systems.

1. Recent models suggest that vaccination is a useful tool for controlling bTB in badgers, with the potential to be applied as an exit strategy from culling. Hence, more detailed investigations of vaccination strategies are required. Field and experimental evidence indicate that the current vaccine (BCG) does not provide complete protection from infection (75), but may confer partial protection, or slow down disease progression. To date most models assume that it confers lifetime protection from infection to a given proportion of the vaccinated population. Technically, these models place vaccinated badgers in a different category that has no increased mortality and no ability to infect others. Therefore, these individuals could become infected, and even react to various live tests, but fail to transmit infection, so the models do not actually assume complete protection, but an inability to become infectious. The available empirical data cannot easily distinguish between a proportion of vaccinated animals being very well protected, and all vaccinated animals experiencing slower disease progression. Such partial protection would lead to a reduced efficacy of disease control and requires further investigation in the field and through modeling. Further evidence is also required to determine the duration of protection (whether complete or partial).
2. Intervention duration and frequency have received little attention in models, and could usefully be explored in more detail. Most models assume either continuous or annual application of management, but recent evidence suggests that breaks in treatment may be possible without significant detrimental effects (54). This is important because even short breaks in management of a single year at a time may reduce overall cost and thus improve the economic outcome.
3. Social perturbation in culled badger populations has so far been simulated using a fixed effect, or by pattern-matching model output with field data. Modeling suggests that the presence of perturbation can be pivotal in determining whether a culling strategy is worth pursuing, but perturbation has only been modeled as an on/off effect. Further empirical evidence on the magnitude of perturbation effects encountered under different conditions, and refined model parameterization are vital to more accurately assess likely outcomes of different culling strategies and allow comparison with other approaches.
4. Within-individual level effects have not been explored in badger models. Where animals are tested, or subjected to management interventions (e.g., vaccination) in stochastic models, independence in outcome is assumed. This means that repeated testing (or repeated vaccination), will eventually detect (or sero-convert) every individual. Instead, it may be that some individuals can never produce a positive test result (or be successfully vaccinated) due to a physiological process/characteristic. This would cause repeated (e.g., annual) management strategies to be less effective, but it is not clear how large such an effect may be.
5. Between-individual effects have not been explored. Most models assume all individuals are the same in terms of their physiological and behavioral responses, although there is clear empirical evidence to the contrary. Social network analyses have revealed individuals occupying different network positions, with associated variation in infection exposure and transmission potential (76). Models that account for individual heterogeneity in transmission rates (within and between species) may be worth investigating with a view to assessing the potential impacts on disease dynamics of removing key individuals in targeted management interventions.
6. Recent interest in selective removal strategies has raised the issue of test performance. In a model the infection status of each individual is perfectly known, whereas test performance determines sensitivity (all infected animals that test negative, regardless of whether latent, infected or infectious). For bTB there is no gold standard test, and thus no way to map an individual onto a simulated categorical state. Thus, test performance is determined globally on the population, and not for each disease state in a model, although empirical evidence suggests some tests have a differential sensitivity according to the stage of disease progression (77, 78). Also, novel probabilistic approaches to describing infection status may help us to incorporate uncertainty in test outcomes and provide a more meaningful way to categorize individuals (79).
7. Theoretical studies have suggested that fertility control may be a useful tool for disease control, particularly in combination with other approaches, but it has yet to be simulated for specific bTB control strategies. Suitable agents are currently available to induce immunocontraception that may last a number of years from a single dose (80) and these are under investigation for badgers, which are unusual in having delayed implantation (http://sciencesearch.defra.gov.uk/Default.aspx?Menu=Menu&Module=More&Location=None&Completed=0&ProjectID=17952).
8. There still appears to be a disconnect between the calculation of R_0_ (close to 1.0), and the high level and lengthy duration of control required to achieve disease eradication in stochastic models. The duration of control is not technically a problem, since R_0_ indicates the level of control required and tells us nothing about the duration. So this disconnect may be because model simulations are not of sufficient duration, or a result of other issues such as the spatial distribution of animals and disease.

### Presenting model outputs to decision makers

It is clear from our experience that some modeling is more informative to decision makers than others. Below we suggest steps to help improve the relevance of modeling to decision makers.

1. It is important to know whether the purpose of the model is to help inform decision making, or to explore the system under study. In the former, the question to be investigated needs to be clearly articulated, ideally with the involvement of decision makers. The question should be specific, with an example graph or table in mind as the output, which allows both parties to agree on the output metric.
2. What the model does and does not include should be agreed with the decision maker. For example, it should be established whether a wildlife bTB model should include cattle so as to estimate changes in herd breakdown rates, or social perturbation arising from the intervention. The model should include all those components that the decision maker regards as important if they are to trust the output, or demonstrate that such components have very limited effect on the output.
3. Models that are well described and identify their assumptions and limitations, are given more weight by decision makers. Mathematical descriptions of model processes may be required for scientific publication, but flow charts are easier to follow. There are also guidelines to present the description of complex individual based models (81, 82).
4. Model description should include details of verification and validation, and some level of sensitivity and uncertainty analysis. Verification is the process of checking that the model does what is expected, and validation is the process of checking output against real world data (where possible). Sensitivity or uncertainty analysis can be used to demonstrate that a decision should be robust to the parameter uncertainty.
5. Model output is often best described in terms of the potential decision, rather than as a prediction of future trends. Models are simplifications, and are unable to capture the future variability of the real world. However, the performance of two modeled strategies will suffer to the same degree from these issues, and so can provide valuable information on their likely relative benefits and hence inform decision making. For the purposes of comparison it may be useful to determine how often one strategy outperforms another, as this will increase confidence in any selection.

These recommendations have applications beyond the bTB/badger system. Specific themes such as those relating to vaccination efficacy, the potential for management interventions to change host behavior and influence disease dynamics in counter-productive ways, and the performance of diagnostic tests are broadly applicable. This illustrates how the body of work on modeling bTB has contributed to our general understanding of the dynamics and management of disease in wildlife hosts and demonstrated how to model these systems.

## Author contributions

All authors listed have made a substantial, direct and intellectual contribution to the work, and approved it for publication.

### Conflict of interest statement

The authors declare that the research was conducted in the absence of any commercial or financial relationships that could be construed as a potential conflict of interest.
